# Comparison analysis of metabolite profiling in seeds and bark of *Ulmus parvifolia*, a Chinese medicine species

**DOI:** 10.1080/15592324.2022.2138041

**Published:** 2022-11-01

**Authors:** MingLong Yin, ChuanRong Li, YuShan Wang, JunHui Fu, YangYang Sun, Qian Zhang

**Affiliations:** a Forestry College, Shandong Agricultural University, Tai’an, China; b Institute of Forest Tree Genetics and Breeding, Taishan Academy of Forestry Sciences, Tai’an, China

**Keywords:** *Ulmus parvifolia*, metabolite profiling, differential metabolites, pharmaceutical value

## Abstract

*Ulmus parvifolia* (*U. parvifolia*) is a Chinese medicine plant whose bark and leaves are used in the treatment of some diseases such as inflammation, diarrhea and fever. However, metabolic signatures of seeds have not been studied. The seeds and bark of *U. parvifolia* collected at the seed ripening stage were used for metabolite profiling analysis through the untargeted metabolomics approach. A total of 2,578 and 2,207 metabolites, while 503 and 132 unique metabolites were identified in seeds and bark, respectively. Additionally, 574 differential metabolites (DEMs) were detected in the two different organs of *U. parvifolia*, which were grouped into 52 classes. Most kinds of metabolites classed into prenol lipids class. The relative content of flavonoids class was the highest. DEMs contained some bioactive compounds (e.g., flavonoids, terpene glycosides, triterpenoids, sesquiterpenoids) with antioxidant, anti-inflammatory, and anti-cancer activities. Most kinds of flavonoids and sesquiterpenes were up-regulated in seeds. There were more varieties of terpene glycosides and triterpenoids showing up-regulated in bark. The pathway enrichment was performed, while flavonoid biosynthesis, flavone and flavonol biosynthesis were worthy of attention. This study identified DEMs with pharmaceutical value between seeds and bark during seed maturation and offered a molecular basis for alternative or complementary use of seeds and bark of *U. parvifolia* as a Chinese medicinal material.

## Introduction

*Ulmus parvifolia* (*U. parvifolia*) is native to China, Japan, and Korea, which is wide-used in landscape.^1^ As a Chinese medicine plant, the bark and leaves of *U. parvifolia* are the main medicinal parts, and are harvested in summer and autumn. In generally, leaves are used when fresh and bark is used when fresh or after drying. The fresh leaves are purported to be an external dressing on wounds and ulcerous tissue. The bark has the value of demulcent, diuretic, expectorant and febrifuge.^2–4^ Additionally, modern medical studies have proved that bark of *U. parvifolia* have the functions of antioxidant, anti-inflammatory,^5^ anti-obesity,^6^ anti-platelet,^7^ anti-cancer,^8^ skin wound healing,^9^ and protection of allergic asthma activities.^10^

Previous studies have provided useful insights into the chemical composition of *U. parvifolia* leaves,^5^ bark,^6–9,11^ and heartwood,^12^ such as flavonoids (e.g., tetra acetyl flavone, isoquercitrin and rutin), sesquiterpenoids (e.g., mansonone C, E, G and santonin), triterpenoids (e.g., oleanolic acid and lupeol caffeate) and steroids and steroid derivatives (e.g., β-sitosterol, stigmasterol, and campesterol). There was very little literatures on the metabolic signatures of *U. parvifolia* seeds. Therefore, in order to make better use of *U. parvifolia*, it is necessary to analyze the differential metabolites in seeds and bark by a credible method.

Metabolomics is a part of systems biology as important as genomics, transcriptomics, and proteomics.^13^ Metabolomics performs qualitative and quantitative analysis of metabolites in biological samples through high-throughput chemical analysis technology, which can directly reflect the life body terminal and phenotypic information.^14^ According to the different application scope, metabolomics fall into targeted metabolomics and untargeted metabolomics. In general, targeted metabolomics focus on only the known metabolites of interest. Whereas, untargeted metabolomics is applied to acquire as many metabolites as possible, and review both known and unknown metabolic changes.^15–17^ The two major analytical techniques used for metabolite fingerprinting are gas chromatography-mass spectrometry (GC-MS) and liquid chromatography-mass spectrometry (LC-MS), while the former approach is widely used to identify volatile metabolites and the latter to detect a wide range of molecules.^18^ It has been reported that the untargeted metabolomics has been applied to analyze the change rules of metabolites in different stages, tissues, and processing modes of different plants.^19–21^ These studies proved that metabolomics analysis could be an authentic tool for comparing active compounds from different organs of *U. parvifolia*.

In this study, the seeds and bark of *U. parvifolia* were collected during seed ripening period. Metabolomic fingerprinting of the two different organs was performed by untargeted metabolomics methods. The purpose of the present study was to detect the metabolic profiles of seeds and bark of *U. parvifolia*, to identify important differential metabolites (DEMs) and key metabolic pathways, and to analyze the biological activities of the differential metabolomics in the two different organs, which would provide theoretical support for developing functional products and clarifying their different pharmacological activities.

## Materials and methods

### Plant materials

**Figure 1.**
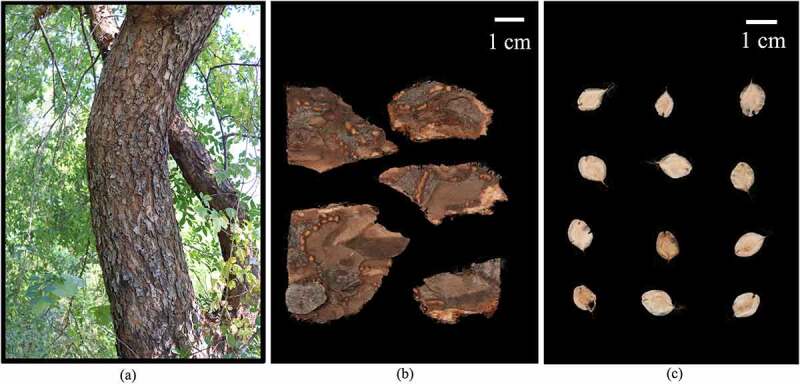
Phenotypes of sampled *U. parvifolia* stem, bark and seeds. (a) Stem. (b) Bark. (c) Seeds. Bar = 1 cm.

In this study, 12 trees were randomly selected from the *U. parvifolia* Germplasm Repository located in the Taishan District of Tai’an, China (36°13′ N, 117°7′ E). The area has a semi-humid continental monsoon climate, with an annual mean temperature of 13.4°C, an extreme minimum temperature of −20.7°C, an extreme maximum temperature of 42.1°C, the annual average precipitation is 678.5 mm, and the frost-free period is 198 days. The soil of the area is brown loam with a thickness between 30 cm and 40 cm, soil bulk density of 1.56 g cm^−3^, the pH value of 5.0 to 5.5, organic carbon content of 6.10 g kg^−1^, total nitrogen content of 0.68 g kg^−1^, and available phosphorus contents of 3.89 mg kg^−1^. Diameter at breast height (DBH) of the selected trees were greater than 20 cm (*Table S1*). Seeds and bark were collected from late October to early November, 2021 (Figure 1). Five grams of seeds from each tree were mixed evenly with six biological replicates. The bark was collected at 1.5 m above the ground, with the size of 10 cm by 10 cm. The bark was cut into small pieces and then well mixed. Each organ had six biological replicates and 12 samples were collected. The samples were frozen immediately in liquid nitrogen, transferred to the laboratory and then stored at −80°C for metabolomics analysis.

### LC-MS sample preparation

80 mg samples, 20 µL internal standard of 2-chloro-L-phenylalanine (0.06 mg mL^−1^) in methanol and 1 mL methanol/water (7/3, *v/v*) were added into 1.5 mL Eppendorf tube with two small steel balls, respectively. Then, the mixture was placed at −20°C for 2 min, ground at 60 Hz for 2 min, extracted with ultrasonic in ice water bath for 30 min and then placed overnight at −20°C. 150 µL supernatants were collected before the mixture was centrifuged at 13,000 rpm at 4°C for 10 min. The extract was filtered through 0.22 µm microfilters, transferred to liquid chromatography vials and stored at −80°C until subsequent LC-MS analysis. Mixed all samples as quality control (QC) samples in the same way as analysis samples.

### LC conditions and MS method

The extracts were analyzed by Dionex U3000 UHPLC system (Thermo Fisher Scientific, Waltham, MA, USA) coupled to a Q exactive orbitrap mass spectrometer (Thermo Fisher Scientific, Waltham, MA, USA). The experimental process referred to the previous study and appropriately modified the parameters in the extraction and separation process.^22^

The LC separation was performed using a ACQUITY UPLC HSS T3 (100 mm × 2.1 mm, 1.8 μm) at a column temperature of 45°C. The flow rate was maintained at 0.35 mL/min. Mobile phase A was water contained 0.1% formic acid (*v/v*) and Mobile phase B was acetonitrile contained 0.1% formic acid (*v/v*). The elution gradient was conducted as follows: 0 min, 5% B; 2 min, 5% B; 4 min, 30% B; 8 min, 50% B; 10 min, 80% B; 14 min, 100% B; 15 min, 100% B; and 15.1 min, 5% B; 16 min, 5% B. Mass spectrometry conditions were performed as follows: ion source: electrospray ionization (ESI) operated in both positive and negative ionization mode; capillary temperature: 320°C; aux gas heater temperature: 350°C; spray voltage: (+3.8, −3) kV; sheath gas flow rate: 35Arb; aux gas flow rate: 8Arb; s-lens RF level: 50; mass range: 100 m/z–1200 m/z; full resolution: 70,000; MS/MS resolution: 17,500.

### Data processing and multivariate analysis

The LC-MS raw data was analyzed by the Progqenesis QI v2.3 software (Nonlinear Dynamics, Newcastle, UK) for extracting characteristic ions of metabolites. Data processing parameters were set as following: precursor tolerance: five ppm, fragment tolerance: 10 ppm, product ion threshold: 5%. The LC-MS data was uploaded to SIMCA software package (version 14.0, Umetrics, Umeå, Sweden) for statistical analysis. The LC-MS data were obtained according to three-dimensional datasets including *m/z*, peak retention time (PRT) and peak intensities, and PRT-*m/z* pairs were used as the identifier for each ion. Metabolite identification was based on Plant Metabolome Database.^23^ Hierarchical cluster heatmap analysis (HCA), principal component analysis (PCA) and (orthogonal) partial least squares-discriminant analysis ((O)PLS-DA) were used to visualize the differential metabolites among the samples. In (O)PLS-DA analysis, variable importance in projection (VIP) and *p*-value can be used to select differentially expressed metabolites (DEMs) in seeds and bark, while the metabolites had the values of VIP > 1, *p-*value < 0.05 and fold change ≥ 2 or fold change ≤ 0.5. HCA and volcano plots were performed in the R software version 4.1.2 (www.r-project.org). Enrichment analysis was performed using MetaboAnalyst (www.Metaboanalyst.ca).

## Results

### Quality control analysis

**Figure 2.**
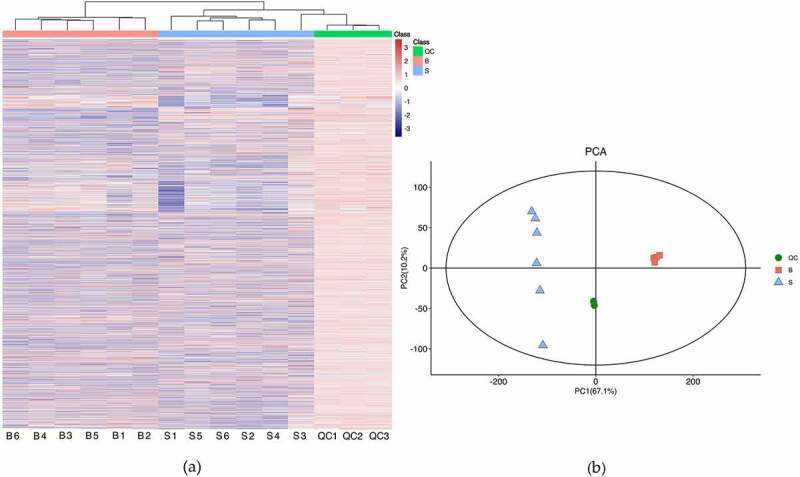
Quality control of samples from biological material. (a) Hierarchical cluster heatmap. (b) Principal component analysis. S and B represent seeds and bark, respectively (the same below). QC represent mixed samples used for quality control.

Six biological replicates for seeds and bark were analyzed by the method of LC-MS, with each replicate consisting of material pooled from 12 trees. The representative base peak chromatogram of the two different part samples were shown in *Figure S1*. Comparing with the base peak chromatogram of mixed samples used for QC with that of these samples from two different organs, the results showed that the response intensity and retention time of each chromatographic peak were basically consistent. The results indicated that the method was reliable and the sample quality was high quality. HCA was conducted with the metabolomics date, which showed that all biological samples from the same part were clustered together, indicating the reliability of the metabolic profiling data (Figure 2a). The result was consistent with PCA analysis, which showed that samples from the two different organs were distributed into two regions. Additionally, the results of QC samples were closely clustered together, indicating that the stability of the instrument was good during the experiment (Figure 2b).

### Metabolic profile of seeds and bark

**Figure 3.**
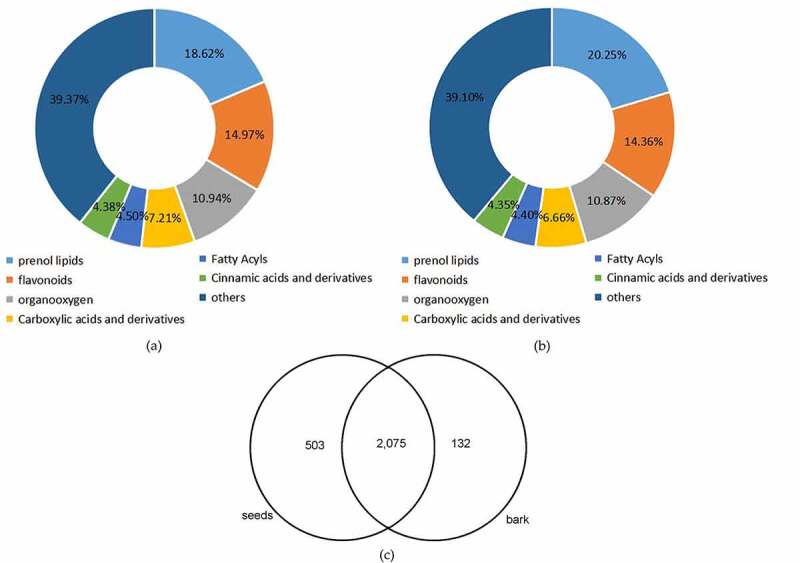
Overview of global metabolic profile in *U. parvifolia* seeds and bark. (a) The classification of metabolites detected in seeds. (b) The classification of metabolites detected in bark. (c) The distribution of metabolites detected in seeds and bark.

A total of 2,710 metabolites were identified in this study, of which 2,578 and 2,207 metabolites were identified through data preprocessing in seeds and bark, respectively (*Table S2*). All the compounds identified in seeds and bark were grouped into 111 and 110 classes, respectively (*Table S3*). As shown in Figure 3a, the maximum class was prenol lipids (480 metabolites, 18.62%) in seed, followed by flavonoids class (386 metabolites, 14.97%). Meanwhile, there were 447 metabolites (20.25%) classified into prenol lipids and 317 metabolites (14.36%) clustered into flavonoids in the bark (Figure 3b). There were considerable common metabolites found in the two different organs. However, there were 503 and 132 components peculiar to the seeds and bark, respectively (Figure 3c and *Table S4*).

### Revealing of differential metabolites in seeds and bark

**Figure 4.**
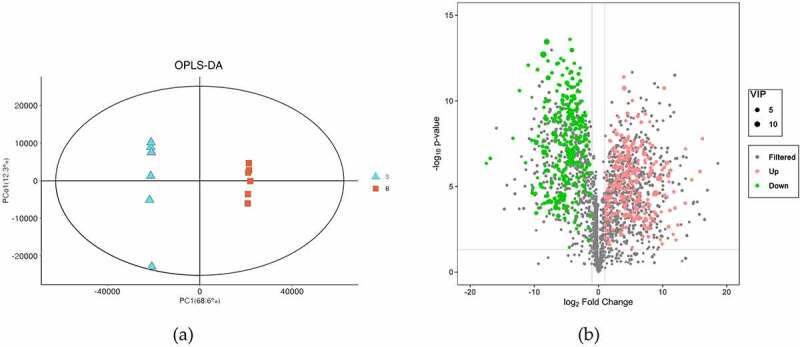
Overview of differential metabolites in *U. parvifolia* seeds and bark. (a) Orthogonal partial least-squares discrimination analysis of the two different organs. (b) Volcano plots depicting the up- and down- regulated metabolites identified between seeds and bark.

To study the global metabolic differences between seeds and bark, (O)PLS-DA was applied as a supervised multivariate method. The result of (O)PLS-DA exhibited good separations between seeds and bark (Figure 4a). In total, 574 DEMs were identified (Figure 4b and *Table S5*). These metabolites were grouped into 52 classes. The most abundant class was prenol lipids (123 metabolites, 21.43%) which was formed as terpene glycosides (35 metabolites), triterpenoids (33 metabolites) and sesquiterpenoids (24 metabolites), followed by flavonoids class (108 metabolites, 18.81%). Flavonoids class mainly included flavonoid glycosides (76 metabolites), flavans (11 metabolites) and biflavonoids and polyflavonoids (8 metabolites).

Through peak area accumulation, the peak areas of DEMs clustered into prenol lipids class in seeds and bark were 2.76 × 10^7^ and 3.10 × 10^7^, respectively. The relative content of the prenol lipids class in seeds and bark were 9.63% and 9.91%. Meanwhile, the peak areas of flavonoids class in seeds and bark were 3.50 × 10^7^ and 4.67 × 10^7^, respectively. The relative content of flavonoids class in seeds and bark were 12.24% and 14.92%, respectively. In addition, flavonoid glycosides subclass was the most important contributor of DEMs enrichment amount in seeds and bark. The relative content of flavonoid glycosides subclass in seeds and bark were 10.50% and 13.44%, respectively.

### Characteristics of flavonoids in seeds and bark

A total of 108 kinds of flavonoids were detected as DEMs between seeds and bark (*Table S6*). Most metabolites in the flavonoids class were formed as flavonoid glycosides subclass. As shown in the HCA, the expression levels of these flavonoids were different in biological materials (*Figure S2*). There were 57 metabolites showing up-accumulated in bark, such as isoquercitrin, naringin, catechin, eriodictin, phlorizin, liquiritin apioside, spinacetin, glucodistylin, trilobatin, etc. In addition, 51 metabolites were up-accumulated in seeds, including avicularin, rutin, myricetion, kaempferol, quercetin, diosmin, sudachiin A, proanthocyanidin A1, procyandin B1, B2, B8, C1, leucopelargonidin, etc. Additionally, 16 and 8 kinds of flavonoids only existed in seeds and bark, respectively. For example, myricetin, quercetin-4’-glucuronide, quercetin 3-*O*-glucuronide, etc. were only found in seeds and 8-glucopyranosylprocyanidin B1, 2”-*O*-Acetylisoorientin, 6”‘-*O*-sinapoylsaponarin, etc. were only found in bark.

### Characteristics of terpene glycosides in seeds and bark

**Figure 5.**
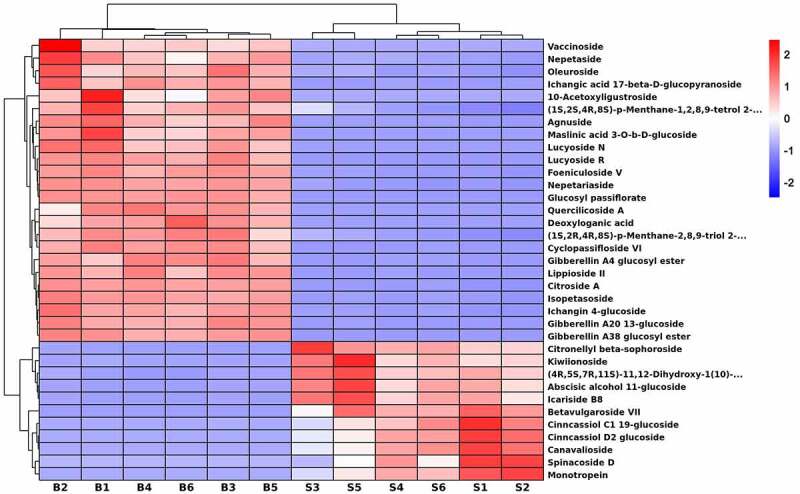
Hierarchical cluster heatmap of terpene glycosides in *U. parvifolia* seeds and bark. S and B represent seeds and bark, respectively.

There were 35 kinds of terpene glycosides expressing different levels between seeds and bark samples. As shown in Figure 5, 24 metabolites were up-regulated in bark, including citroside A, vaccinoside, nepetaside, oleuroside and agnuside, etc. Additionally, 11 metabolites were enriched in seeds, such as spinacoside D, cinncassiol D2 glucoside, betavulgaroside VII, monotropein, icariside B8. In addition, there were 3 and 2 unique metabolites identified in seeds and bark, respectively. For example, betavulgaroside VII, cinncassiol D2 glucoside and spinacoside D only existed in seeds and lucyoside R and maslinic acid 3-*O*-b-D-glucoside only existed in bark.

### Characteristics of triterpenoids in seeds and bark

**Figure 6.**
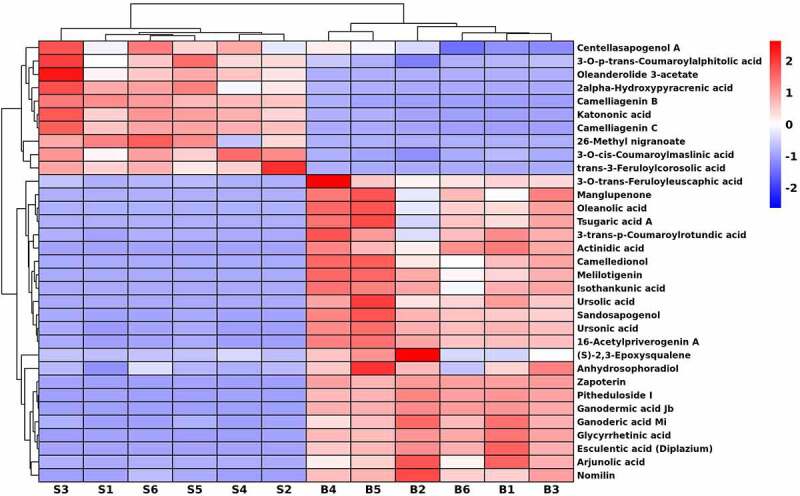
Hierarchical cluster heatmap of triterpenoids in *U. parvifolia* seeds and bark.

To evaluate characteristics of metabolites in the triterpenoids class from different fractions of *U. parvifolia*, the identified 574 DEMs were analyzed. As shown in Figure 6, a total of 33 kinds of triterpenoids were detected between seeds and bark samples. Among them, centellasapogenol A, oleanderolide 3-acetate, katononic acid, 3-*O*-p-trans-coumaroylalphitolic acid, 2alpha-Hydroxypyracrenic acid, camelliagenin B, camelliagenin C, 26-methyl nigranoate, trans-3-feruloylcorosolic acid and 3-*O*-cis-coumaroylmaslinic acid were up-regulated in seeds compared with bark. Twenty-three metabolites were up-regulated in bark, such as 3-*O*-trans-feruloyleuscaphic acid, manglupenone, oleanolic acid, tsugaric acid A, 3-trans-p-coumaroylrotundic acid, arjunolic acid, camelledionol, melilotigenin, isothankunic acid, ursonic acid, sandosapogenol, ursolic acid, esculentic acid (diplazium), 16-acetylpriverogenin A, (S)-2,3-epoxysqualene, anhydrosophoradiol, zapoterin, pitheduloside I, ganodermic acid Jb, ganoderic acid Mi, glycyrrhetinic acid, and nomilin.

### Characteristics of sesquiterpenoids in seeds and bark

**Figure 7.**
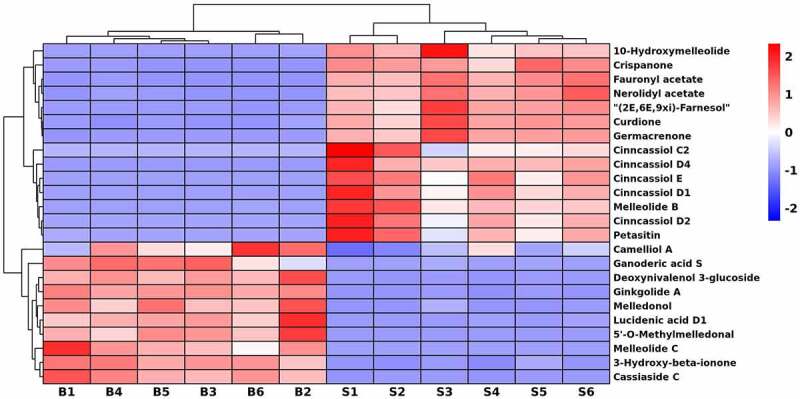
Hierarchical cluster heatmap of sesquiterpenoids in *U. parvifolia* seeds and bark.

In this study, there were 24 DEMs involved in sesquiterpenoids (Figure 7), of which 14 metabolites were up-accumulated in seeds, e.g., curdione, cinnassiol C2, D1, D2, D4, E, 10-hydroxymelleolide, crispanone, nerolidyl acetate, fauronyl acetate, (2E,6E,9xi)-farnesol, germacrenone, melleolide B and petasitin. Among them, cinncassiol D2 and D4 were only found in seeds. In addition, 3-hydroxy-beta-ionone, ginkgolide A, cassiaside C, camelliol A, ganoderic acid S, deoxynivalenol 3-glucoside, melledonol, lucidenic acid D1, 5’-*O*-methylmelledonal and melleolide C were enriched in bark.

### Major differential metabolic pathways in seeds and bark

**Figure 8.**
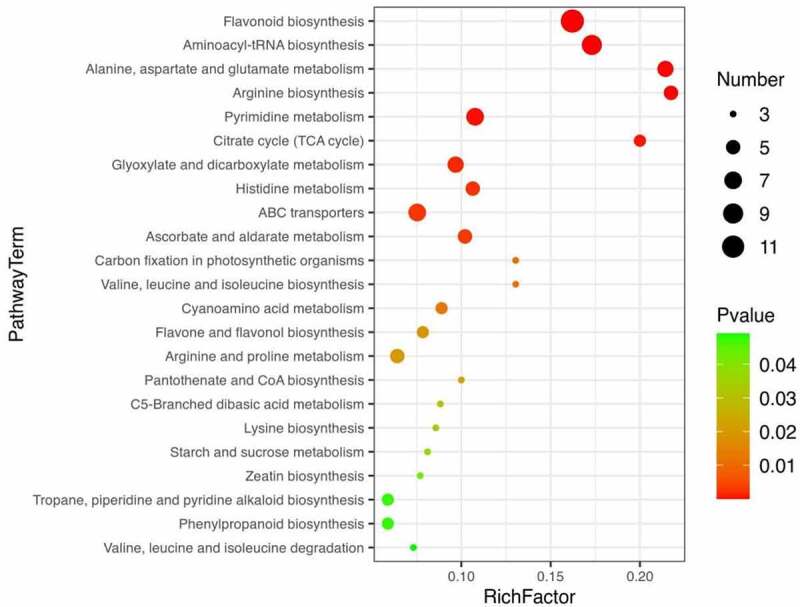
Bubble map of metabolic enrichment pathways. Enrichment of differential metabolites of seeds and bark in KEGG pathways.

Based on DEMs identified between seeds and bark, Kyoto Encyclopedia of Genes and Genomes (KEGG) (http://www.genome.jp/kegg/) was used for metabolic pathway enrichment analysis (*Table S7*). As Figure 8 shown, a total of 23 metabolic pathways were statistically significant (*p*-value < 0.05). Among them, flavonoid biosynthesis (*Figure S3*) and flavone and flavonol biosynthesis (*Figure S4*) are particularly worthy of our attention. Because 12 and 4 metabolites in the flavonoids class were involved in both pathways, respectively. As shown in the flavonoid biosynthesis pathway, caffeoylquinic acid, caffeoylshikimic acid, leucopelargonidin, quercetin, and myricetin were up-regulated in the pathway. In addition, quercetin, myricetin, and rutin were up-regulated in the flavone and flavonol biosynthesis pathway.

## Discussion

In this study, a total of 2,578 and 2,207 metabolites were identified in seeds and bark, respectively. Seeds contained more kinds of compounds than bark. There were more compounds in prenol lipids class in seeds than bark. The same was true for flavonoids class, organic oxygen compounds class and carboxylic acids and derivatives class. By peak area accumulation, the flavonoids class was the most important contributor of DEMs enrichment amount in seeds and bark, followed by prenol lipids class. The results may be due to the conversion of nutrients into large molecules (e.g., soluble sugar and starch, lipid, amino acids, soluble protein, anthocyanidin) which are not easily breakdown during seeds development.^24,25^ Meanwhile, there were 503 and 132 unique metabolites found in seeds and bark, respectively. For example, hirsutin, sinapine, 4-hydroxyphthalide and frenolicin B were found to be expressed only in seeds of *U. parvifolia*, while isothankunic acid, colubrinic acid and medicagenic acid were found to be unique metabolites of bark. The result indicated that seeds and bark may have special pharmacological activities, respectively. Of course, these unique metabolites were extracted and analyzed during seeds maturation period, but our results provided a basis for future experiments.

The analysis of PCA and (O)PLS-DA were applied to better evaluate the differences between seeds and bark. As a result, 574 DEMs were identified between two organs, which were grouped into 52 classes, including prenol lipids class, flavonoids class, organooxygen compounds class, carboxylic acids and derivatives class. Among them, prenol lipids class contained terpene glycosides (e.g., monotropein, agnuside), triterpenoids (e.g., oleanolic acid, ursonic acid), sesquiterpenoids (e.g., ginkgolide A, curdione), etc. Flavonoids class were mainly composed of flavonoid glycosides (e.g., isoquercitrin, rutin), *O*-methylated flavonoids (e.g., citromitin), flavans (e.g., catechin), flavones (e.g., myricetin), etc.

Flavonoids exist ubiquitously in plants (flowers, leaves, bark, and seeds) and more than 8,000 different compounds have been identified.^26^ Such as, 653 flavonoid metabolites were identified from leaves and seeds of ginko at two developmental stages.^27^ Flavonoids mainly combined with glycosides to form flavonoid glycosides in plants rather than in free form,^28^ which was consistent with the present study. Previous research has shown that flavonoids had diverse pharmacological effects on a variety of diseases such as cancer, cardiovascular disease, microbism, diabetes and HIV.^29^ The research by Lee et al. proved that the extract of *U. parvifolia* bark have anti-obesity properties and catechin may be the main active ingredient in the extract.^6^ Additionally, kaempferol derivatives in the extract of *U. parvifolia* bark have showed significant curative effect on hepatitis C viruses.^8^ In the present study, catechin and kaempferol derivatives were identified. One of the interesting things we found was that catechin was only existed in bark and rutin was only existed in seeds, but the medicinal effects of catechin and rutin were similar. Both compounds had shown significant anti-cancer and anti-inflammatory activities.^30,31^ Compounds of glucodistylin, procyandin B1, B2, B8 and leucopelargonidin, expressing different levels in bark and seeds, had obvious inhibitory effect on diabetes.^32–34^ Besides, trilobatin enriched in bark displayed synergistic anti-HIV activity, combined with other antiretroviral agents.^35^ The above information indicates that alternative use of seeds and bark can be considered.

Terpene glycosides belong to the class of organic compounds known as prenol lipids. These compounds were widely found in nature and comprised a great variety of different structures due to the individual aglycones or carbohydrates.^36^ To date, the distribution of terpene glycosides within different organs of *U. parvifolia* was much less well-documented. Terpene glycosides had attracted much attention because these compounds had inhibited oxidative damage, anti-inflammatory and wound healing effects. Besides, some terpene glycosides also could be used as flavor precursors and detergents.^37^ We found that citroside A and agnuside were up-regulated in bark which could inhibit inflammation.^38,39^ Additionally, the compound of monotropein could accelerate wound healing, which was up-regulated in seeds.^40^ According to the information, the bark could be applied to inhibit inflammation, and seeds had better effect on wound healing than the bark. However, further research is needed to determine whether seeds can be used in the form of traditional Chinese medicine.

As previous studies reported, triterpenoids have anti-inflammatory, anti-cancer, anti-viral, antioxidant and anti-diabetic activity.^41^ Such as oleanolic acid,^42^ arjunolic acid,^43^ melilotigenin,^44^ ursonic acid,^45^ and ganoderic acid,^46^ which have significant anti-cancer and anti-inflammatory effects. These compounds were up-regulated in bark. Katononic acid and centellasapogenol were up-regulated in seeds which had been verified their potential as inhibitor drugs for diabetes therapies.^47,48^ In addition, lupeol caffeate and ulmicinD were not identified, perhaps only existed in leaves of *U. parvifolia*. There were few reports on triterpenoids from seeds of *U. parvifolia*. Our study showed a comprehensive profile of triterpenoids in seeds and bark. In addition, we found that the number of triterpenoids in the two different organs of *U. parvifolia* were almost the same, and the most up-regulated triterpenoids were in bark. According to the information, we suggest that bark may have potential roles in the treatment of cancer and inflammation, and seeds can be used as raw material for anti-diabetic drugs.

Sesquiterpenes are a class of 15-carbon isoprene compounds that exist naturally in higher plants, microorganism and marine life.^49^ Sesquiterpenoids provided encouraging leads for cytotoxic, antioxidant, anti-inflammatory, anti-bacterial and other activities.^50^ For example, curdione and ginkgolide A were reported to have potential effects on anti-inflammatory, anti-tumor, neuroprotective and anti-atherosclerosis effects.^51,52^ In addition, curdione, up-regulated in seeds, could be used to inhibit idiopathic pulmonary fibrosis (IPF).^53^ Cinnassiol D1 enriched in seeds that had the immunomodulatory activity.^54^ However, santonin was not been found, may be only derived from leaves. In this study, we found that there was only a slight difference in the amount of sesquiterpenoids contained in the two different organs of *U. parvifolia*. Based on the information, we can infer that seeds may have some similar medicinal effects as bark.

Pathway enrichment analysis based on metabolites can provide insight into the mechanism of metabolic pathways in different samples.^55^ The biosynthesis of flavonoids is complex, which occurs at the junction of the shikimate pathway (polyketide pathway) and acetate pathway under the catalysis of a series of key enzymes (e.g., CHS, CHI, FLS).^56,57^ In this study, we should focus on flavonoid biosynthesis and flavone and flavonol biosynthesis. As shown in the two pathways, quercetin, myricetin, and rutin were up-regulated in both pathways. As mentioned earlier in this article, quercetin, myricetin and rutin have the beneficial effects on inflammation, immunity, diabetes, atherosclerosis and thrombosis. Meanwhile, the three compounds were up-regulated in seeds. The results suggest that seeds of *U. parvifolia* may have better function of treatment for diabetes, inflammation, atherosclerosis, and thrombosis, comparing with bark of *U. parvifolia*.

## Conclusions

In this study, metabolite profiling, characteristics of DEMs and pathway enrichment were performed to evaluate the pharmaceutical value of seeds and bark of *U. parvifolia*. A total of 2,578 and 2,207 metabolites were identified in seeds and bark, respectively. Additionally, 574 DEMs were detected in the two different organs of *U. parvifolia*, including prenol lipids class, flavonoids class, etc. The main DEMs in the seeds and bark were further analyzed, including flavonoids, terpene glycosides, triterpenoids and sesquiterpenoids. Enrichment of metabolics pathway was performed, we focused on flavonoid biosynthesis and flavone and flavonol biosynthesis. We can draw the following conclusion: The bark and seeds of *U. parvifolia* can be alternative or complementary utilized to develop potential drugs for the treatment of tumors, diabetes, inflammation, atherosclerosis and thrombosis. However, this was a preliminary study as samples were collected only during seeds maturation stage. Further studies are needed to analyze the changes of metabolites in different organs according to developmental stages and to confirm methods for proper use of active components.

## Supplementary Material

Supplemental Material
