# Supplementary figures and images for: Comparison analysis of metabolite profiling in seeds and bark of Ulmus parvifolia, a Chinese medicine species
Source: Plant Signal Behav. 2022 Nov 1;17(1):2138041. doi: 10.1080/15592324.2022.2138041 (PMC9629078; doi:10.1080/15592324.2022.2138041)

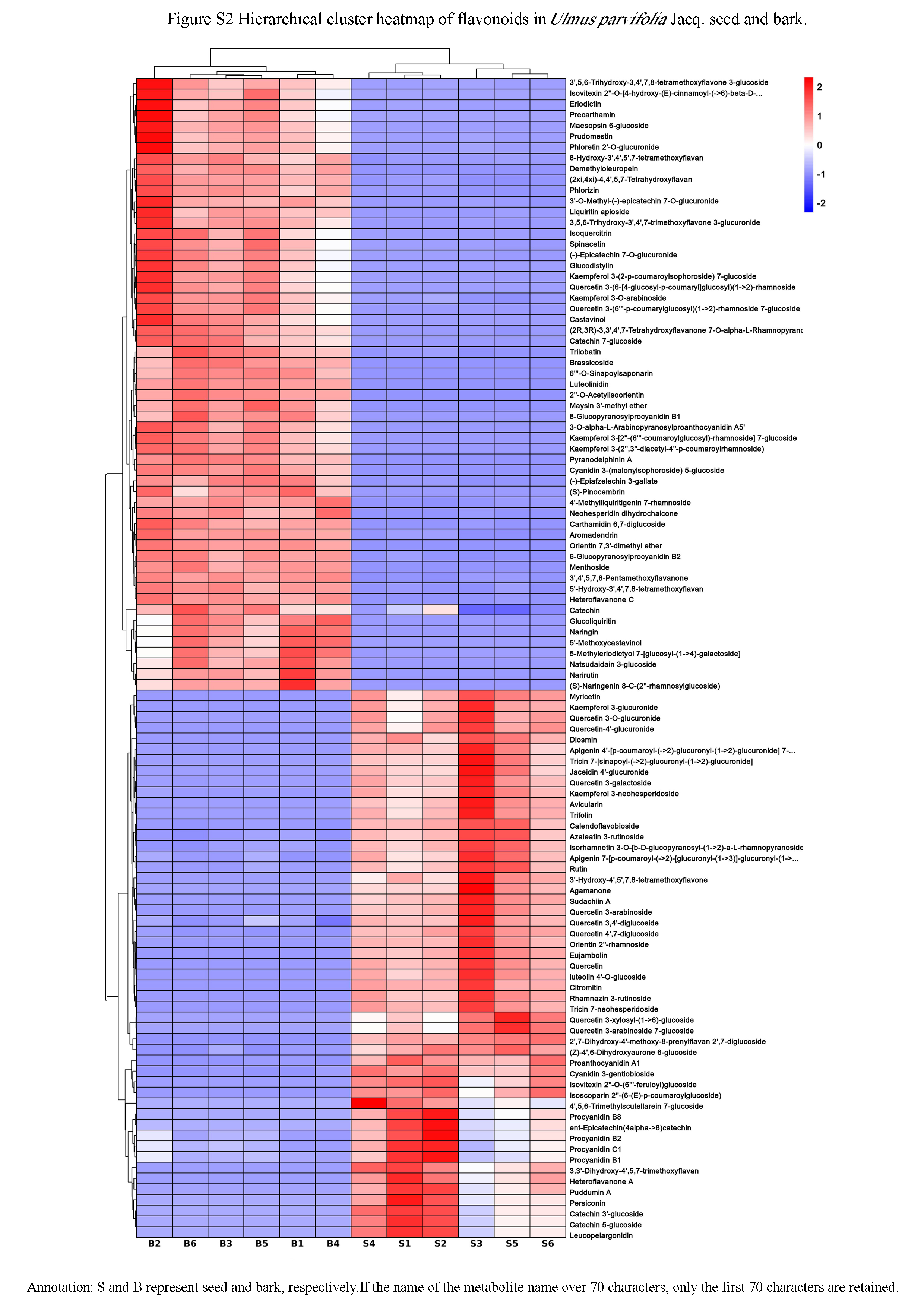

Supplement: Supplemental Material [file KPSB_A_2138041_SM4697.zip › Figure S2.jpg]

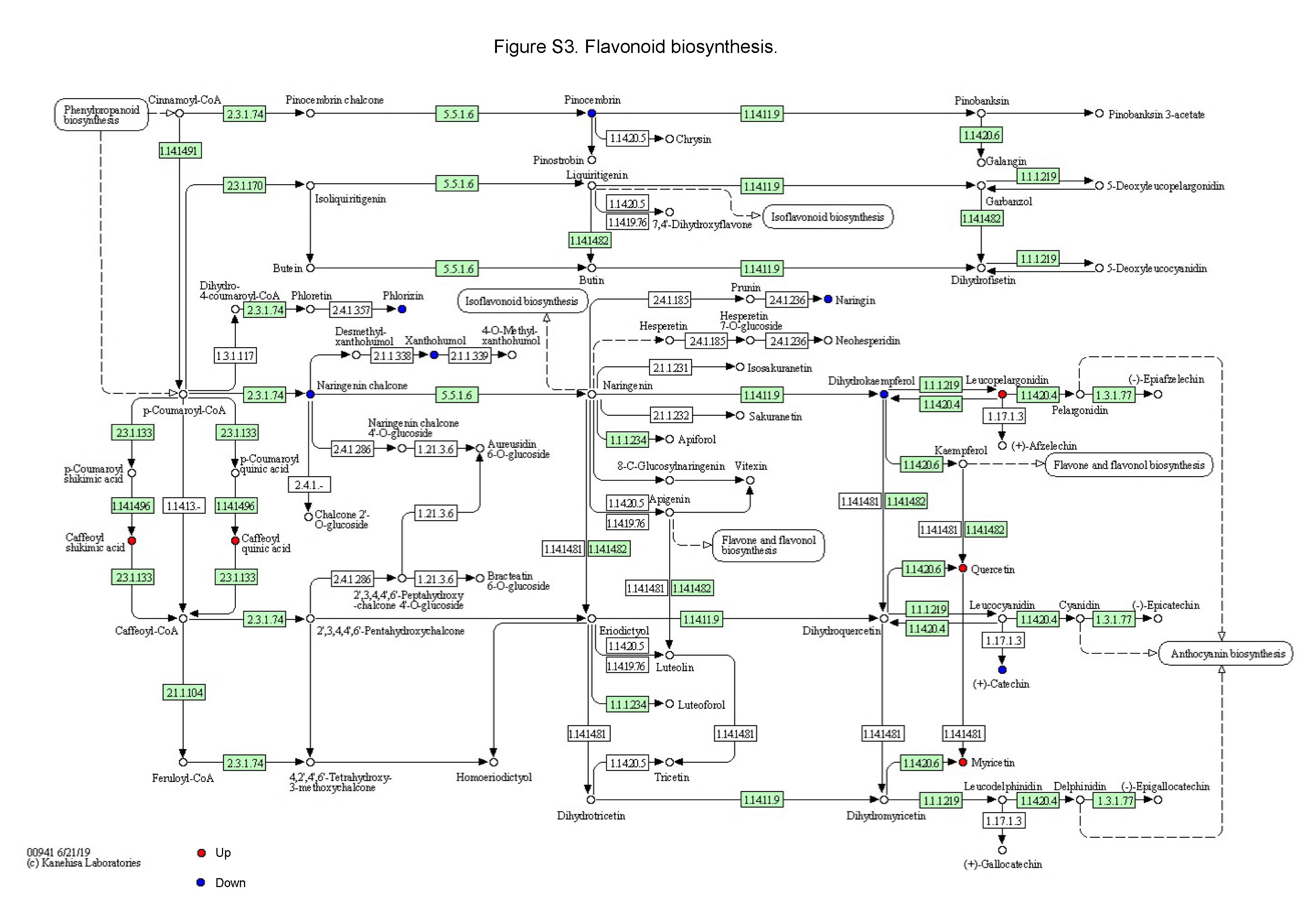

Supplement: Supplemental Material [file KPSB_A_2138041_SM4697.zip › Figure S3.jpg]

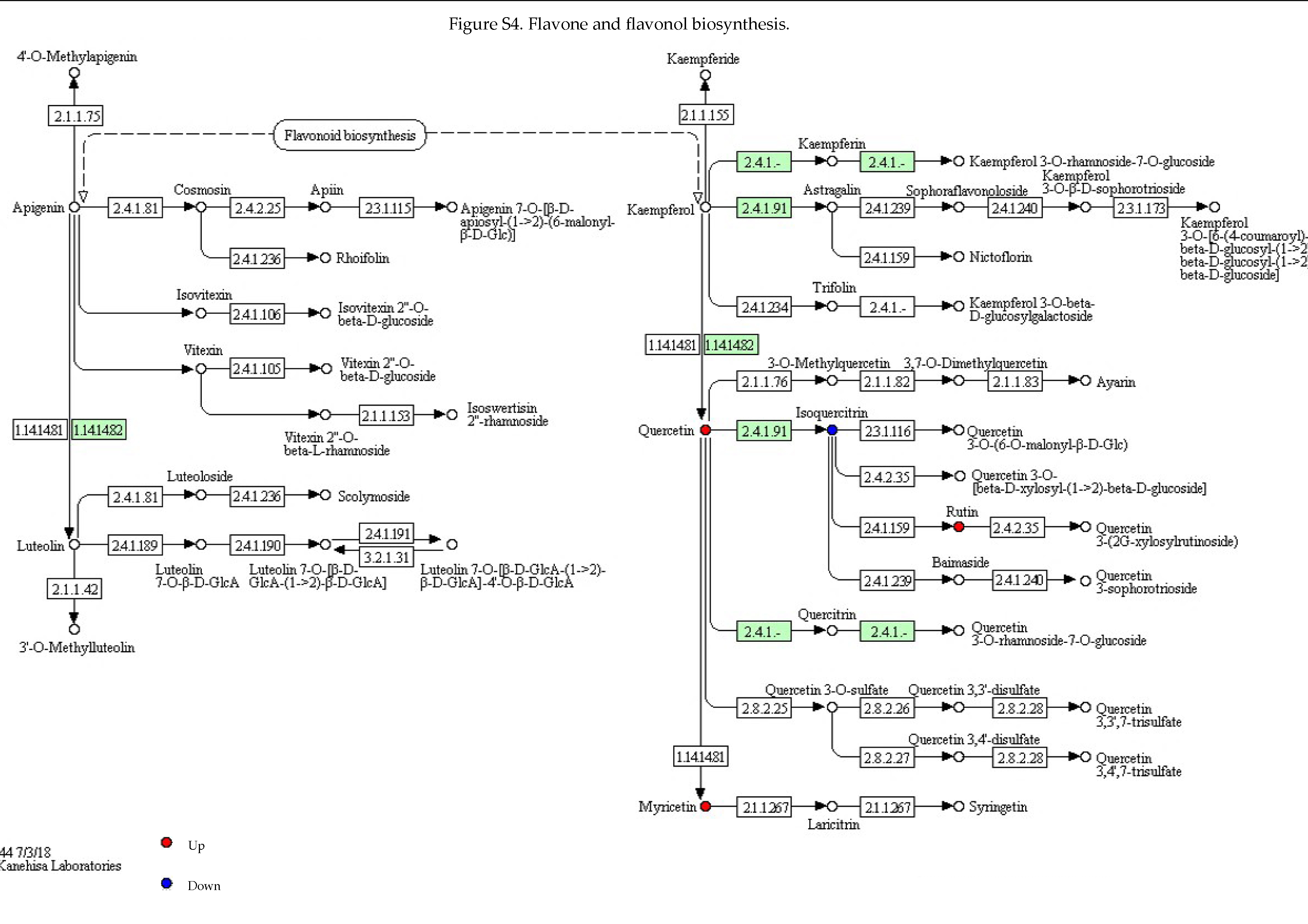

Supplement: Supplemental Material [file KPSB_A_2138041_SM4697.zip › Figure S4.jpg]
