# Supplementary material for: Comparison analysis of metabolite profiling in seeds and bark of Ulmus parvifolia, a Chinese medicine species
Source: Plant Signal Behav. 2022 Nov 1;17(1):2138041. doi: 10.1080/15592324.2022.2138041 (PMC9629078; doi:10.1080/15592324.2022.2138041)
Supplement: Supplemental Material [file KPSB_A_2138041_SM4697.zip › Figure S1.doc]

Figure S1. Base peak chromatogram (in positive and negative ion modes) of seeds(S1-S6) and bark(B1-B6), respectively.

Positive ion modes were showed as following:

Negative ion modes were showed as following:
